# A systematic review of collaborative robots for nurses: where are we now, and where is the evidence?

**DOI:** 10.3389/frobt.2024.1398140

**Published:** 2024-06-05

**Authors:** Grace Titilayo Babalola, Jenna-Marie Gaston, Joseph Trombetta, Stephanie Tulk Jesso

**Affiliations:** ^1^ Department of Systems Science and Industrial Engineering, SUNY Binghamton, Binghamton, NY, United States; ^2^ Human-Centered Mindful Technologies Lab, SUNY Binghamton, Binghamton, NY, United States

**Keywords:** robot, cobot, technology, evaluation, nursing, healthcare

## Abstract

**Introduction:** Robots present an opportunity to enhance healthcare delivery. Rather than targeting complete automation and nurse replacement, collaborative robots, or “cobots”, might be designed to allow nurses to focus on high-value caregiving. While many institutions are now investing in these platforms, there is little publicly available data on how cobots are being developed, implemented, and evaluated to determine if and how they support nursing practice in the real world.

**Methods:** This systematic review investigates the current state of cobotic technologies designed to assist nurses in hospital settings, their intended applications, and impacts on nurses and patient care. A comprehensive database search identified 28 relevant peer-reviewed articles published since 2018 which involve real studies with robotic platforms in simulated or actual clinical contexts.

**Results:** Few cobots were explicitly designed to reduce nursing workload through administrative or logistical assistance. Most included studies were designed as patient-centered rather than nurse-centered, but included assistance for tasks like medication delivery, vital monitoring, and social interaction. Most applications emerged from India, with limited evidence from the United States despite commercial availability of nurse-assistive cobots. Robots ranged from proof-of-concept to commercially deployed systems.

**Discussion:** This review highlights the need for further published studies on cobotic development and evaluation. A larger body of evidence is needed to recognize current limitations and pragmatic opportunities to assist nurses and patients using state-of-the-art robotics. Human-centered design can assist in discovering the right opportunities for cobotic assistance. Committed research-practice partnerships and human-centered design are needed to guide the technical development of nurse-centered cobotic solutions.

## 1 Introduction

As the Industry 4.0 movement has expanded beyond typical manufacturing environments, there has been growing enthusiasm for robotics and automation in healthcare over the past few decades, with the goal of enhancing efficiency, reducing costs, and improving patient outcomes (Dario et al., 1996; IBIS World, 2023). Collaborative robots, also known as “cobots”, are designed to work collaboratively with humans in a shared workspace and have many benefits in comparison to traditional industrial robots, including higher productivity, decreased labor costs, flexibility, improved quality, and enhanced workspace (Colgate et al., 2002; Couroussé and Florens, 2014). Because robotic technology has high repeatability and accuracy, a major advantage to incorporating cobots into new settings is the potential to eliminate a substantial amount of human error, which accounts for approximately 90% of all industrial and system failures (Senders and Moray, 2019).

Though the earliest robots in healthcare were introduced in the 1980s and focused on surgical applications (Dobbs et al., 2017; Goh and Ali, 2022; Gamal et al., 2024), robots developed for healthcare today cover a much broader array of capabilities including disinfection (Kaiser et al., 2020), prescription drug dispensing (Yadav et al., 2022), physical therapy assistance (Karabegović et al., 2021; Anson et al., 2023), emotional support (Morgan et al., 2022), and social companionship (Lorenz et al., 2019; Singla and Nguan, 2022). Others note potential concerns, including challenges with clinical workflow integration, technical limitations of robots, and risks of over-automation reducing human interactions (Elish, 2020; Jabr and Sandhu, 2020; Lawrence et al., 2022; Klebbe et al., 2023). With the growing appetite for cobots, it is important to evaluate their potential impacts on human collaborators, including patients and staff. In particular, while nurses play a central caregiving role and comprise the largest segment of the healthcare workforce worldwide, nursing shortages (Bourgault, 2022; Ford and Thareja, 2023), burnout (Dall’Ora et al., 2020; Lieneck et al., 2023), and workload overload (Pérez-Francisco et al., 2020; Rotenstein et al., 2023) may explain why many robotic and cobotic applications are being proposed to either assist or replace nurses (Buerhaus, 2018; Kangasniemi et al., 2019).

The purpose of this systematic review is to investigate how cobots are being designed and used to assist nurses within hospital settings, as well as how they are implemented and evidence of their effectiveness *in-situ*. We intend to examine available peer-reviewed evidence to provide a comprehensive understanding of the types of cobotic capabilities that are currently being developed, are market-ready, or are in use in healthcare, and to compile evidence on how cobots affect nursing work and patient care. A primary motivation for this review is due to our awareness of multiple cobotic platforms such as TUG Automated Robotic Delivery System by Aethon Inc. (Summerfield et al., 2011; Aethon Inc, 2018; Teng et al., 2022) and Moxi service robot by Diligent Robotics Inc. (Tietze and Mcbride, 2020; Braker et al., 2021; Diligent Robotics, 2023), which are already being implemented into healthcare systems in the United States for at least 5 years, and our inability to find peer-reviewed scientific evidence on their usage and effectiveness. Insights provided from this review can help inform effective evaluation and adoption of approaches that maximize the benefits of healthcare cobots; it is also critical to proactively assess technological limitations and knowledge gaps to promote an evidence-based approach to technology investment.

In this work, we are less concerned with tools which are becoming available and implemented as we are interested in finding and presenting evidence of effectiveness and user perceptions of cobots after actual human interactions in lab or clinical environments. This review builds upon prior works which survey existing robotic platforms and consider future implications (e.g., Kangasniemi et al., 2019; Christoforou et al., 2020; Persson et al., 2022) by taking a detailed look at original research involving and which provide the broader community with actual evidence of how humans interact with such tools, their technological readiness, and how they can affect nursing work and patient care. Prior reviews have examined the general use of robots and automation in nurses’ work with the majority focusing on caregivers’ perceptions of robots in healthcare, patients’ utilization of robots, and physical activity (Persson et al., 2022). While some reviews focused on a particular type of robot, such as socially assistive anthropomorphic or zoomorphic robots in healthcare facilities (Papadopoulos, 2018), others took a wide view of the use of robotic technology in clinical domains such as the care of elderly people (Leonardsen et al., 2023). Kangasniemi et al. (2019) reviewed the utilization of robots and other automated devices by nurses from 2010 - 2018, focusing on findings that promote good work routines and health outcomes for the patients, and Persson et al. (2022) conducted a scoping review on caregiver’s use of robots focusing on their effect on work environment. Though these reviews provided important insights, major advances in artificial intelligence, machine learning, and robotics in recent years have enabled new functionalities and applications relevant to nursing (Lu et al., 2021), coupled with new enthusiasm for cobots necessitates the thorough examination described in this review. Our intention is to provide an updated review of literature which offers references to recent literature which actually provides evidence on the true state of the use and effectiveness of hospital cobots which are intended to aid nurses.

## 2 Methodology

**FIGURE 1:**
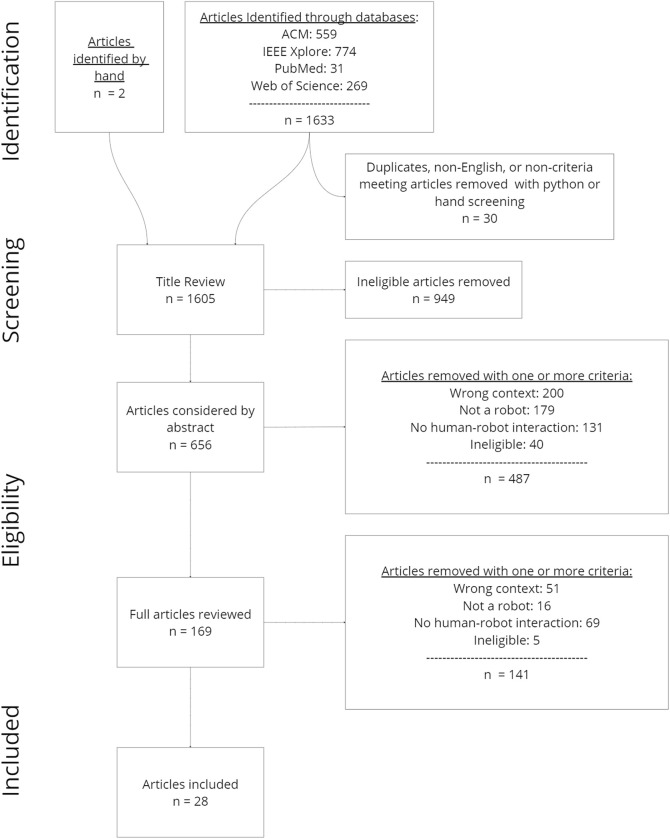
Systematic review process and numbers of articles included and excluded. Articles were first identified through online databases, and then manually pre-processed using initial exclusion criteria. Next, all articles were manually reviewed by title and abstract before reviewing full articles to evaluate which articles were included.

This systematic literature review was conducted in several stages following PRISMA guidelines for systematic review (see Figure 1). This review aims to provide a broad and thorough examination of how cobots are being designed, implemented and used to assist nurses within hospital settings, and the impacts they have on nurses and the patients they care for.

### 2.1 Identification and screening

A systematic database and journal search was included in the identification of relevant literature for this review. The journals included are ACM, IEEE Xplore, Pubmed, and Web of Science. This selection allowed for a wide variety of healthcare, robotic, and robot-human interaction content, relevant to the aim of this review. The articles that went through the systematic review were retrieved on 29 December 2022, and were published in January 2018 onwards, with the main objective of the search being to look for articles that describe robot applications meant to collaborate with nurses (as a primary user, or as a secondary user) within a hospital setting. The exclusion criteria for this retrieval were to exclude articles not written in English, non-peer reviewed (e.g., dissertations), not original research (e.g., other literature reviews), and published prior to 2018 (see Table 2). From this search, 1,859 (1603 from title review +256 excluded articles that were considered irrelevant based on the retrieval exclusion criteria) articles were collected. This includes articles that must have the terms robot or cobot, hospital, and some permutation of nurse (e.g., nursing, nurse, nurses), and must be written after 2018 (see Table 1). From this preliminary batch of articles, *n =* 256 were excluded for being a duplicate (*n* = 29), published before 2018 (*n* = 143), or being identified as irrelevant records which were not related to actual literature (*n* = 84). From here, *n =* 1603 articles were included in the title review phase. The two categories used to identify the article based on its title are shown in Table 1.

**TABLE 1:** Two categories of search criteria used to identify articles.

Robot terms	“Robot” and “cobot”
Clinical domain terms	“hospital” and “nurs*” (e.g., nursing, nurse, nurses)

### 2.2 Consensus and eligibility

#### 2.2.1 Title review

Articles that made it past the initial screening were listed in a spreadsheet which included pertinent details for review as well as references to the articles. First, raters individually reviewed the titles of each article and excluded articles that were easily identifiable to the researchers as irrelevant to the purpose of this review. Criteria used to remove irrelevant titles included, (1) non-peer-reviewed (e.g., dissertations), and not original research (e.g., other literature reviews) articles, (2) duplicate articles (3) articles before 2018 (4) articles not written in English. Beyond this, raters removed articles which were obviously not related to healthcare and which did not feature a robot intended for human interaction. Raters were intentionally conservative in ratings such that articles which were deemed possibly irrelevant were still included for abstract review to reduce the possibility of inadvertently removing relevant articles.

After titles were reviewed, *n* = 949 articles were excluded and *n* = 656 eligible articles were reviewed further by their abstracts.

#### 2.2.2 Abstract review

The abstracts were retrieved by hand and sorted in a shared sheet for review by the group. During the abstract review, a set of exclusion criteria was used to screen whether the content of the article was relevant for the purpose of this systematic review. Three independent raters manually reviewed the abstracts of the articles of the screened articles. A table of the abstract exclusion/inclusion criteria is shown below in Table 2 alongside their respective codes. Articles were excluded if (1) the robot was used in the wrong context, including environments outside of the hospital or for purposes outside of healthcare entirely, (2) if the study featured a technology which did not meet our definition of a robot. We defined a robot as a physically embodied mechanical device with autonomous features. We also excluded surgical robots and autonomous wheelchairs because they were not relevant to our research goals. We also excluded (3) articles which did not involve actual human-robot interactions, either because the robot was not intended for direct human interaction, or because the study only involved hypothetical interactions with a robot, such as an opinion survey. The human interaction also needed to be with a nurse or with a patient as a means of reducing the nurses’ workload (e.g., delivering medications to patients). Finally, we excluded (4) articles which were ineligible using the title review criteria.

**TABLE 2:** Evaluation criteria used for inclusion and exclusion.

LOC = location of care	All studies must relate to robots targeted to a physical healthcare setting (i.e., a hospital)
NR = Not a robot	A robot is defined as: a physically embodied mechanical device with autonomous features
R = Irrelevant	Anything which we could not screen from the title review, but is still irrelevant, including articles not written in English, non-peer reviewed (e.g., dissertations), not original research (e.g., other literature reviews), and published prior to 2018; AND no surgical robots
NHI = No human interaction	No human interaction. EITHER robot is not intended to work with humans in some way, such as robots that are intended to passively sense or operate in the background; OR study does not include any data or discussion of Human-Robot Interactions, where clinicians/patients interact with the robot, such as descriptions of engineering efforts, validation of control algorithms, or surveys of opinions

The raters independently examined the abstracts from about 4.6% of the papers (*n* = 30 articles) to gauge consensus. The first agreement on the articles to include and exclude was 67%. After this initial review, the raters reviewed a portion of the articles that had been coded together and discussed the rationale behind each coding choice in order to reach a better consensus. Following this exercise, each rater independently reevaluated the initial 30 article abstracts using their own and their co-rater’s codes which yielded an 80% consensus (agreement) on articles to include. In cases in which raters disagreed, the senior author (STJ) examined abstracts to make a final determination of inclusion. The remaining articles (*n* = 626) were randomly divided between the raters to review independently. *N* = 487 articles were excluded for not meeting inclusion criteria (in which articles could be excluded for more than one reason), and *n* = 169 articles were taken into consideration for a complete article evaluation.

#### 2.2.3 Full article review

The remaining *n* = 169 articles were divided between the three raters during the full article review process, and eligibility was decided based on the same inclusion/exclusion criteria listed in Table 2 as well as additional criteria which include, excluding studies wherein (1) the usage of collaborative robots is in other healthcare settings (e.g., rehabilitation centers, nursing homes, etc.) other than a hospital, and (2) studies that do not include physical robots like survey papers. After a full review of the 169 articles, a total of *n* = 141 articles were eliminated, leaving a total of *n* = 28 articles that were included.

### 2.3 Data extraction and analysis

In this process, the rater read the article and filled out a Google form designed to collect and arrange the data that was deemed to be relevant to the intention of this review. The Google form’s entry fields were iteratively modified as the articles were read in order to capture and compare the most relevant details, leading to a reevaluation and re-categorization of the data for holistic understanding. The final dataset included information on the expressed motivation for the design effort, the target primary users (e.g., nurses, patients, more broadly clinical staff, elderly people, etc.), country in which the research was conducted, the robot product that was created or applied in the study (e.g., “Pepper” from United Group Robotics Boumans et al., 2019), the application (e.g., social robots for emotional support or companionship, service or logistics), cobot functionalities and features (e.g., mobile base, functional arm(s), charging base), robot morphology (i.e., the physical form of the cobot, including anthropomorphic, or humanlike; zoomorphic, or animallike; or mechanistic), design sample size, and technology readiness level (Manning, 2023), which was included in the final dataset presented in the “Results” section.

## 3 Results

### 3.1 Reviewed articles and product matrix

Our intention was to look at publications that highlighted the types of cobot platforms that were in the process of being developed or were currently being used for in-patient healthcare. Because our final dataset only included robot platforms which are intended for direct human interaction and assistance to humans in some way, we consider all included applications as “cobots”, whether or not they are labeled as such by authors. We also examined the forms and functionalities of these cobots, intended uses, and market-readiness in order to understand how cobots will affect nursing work and patient care. The 28 selected articles were written by authors who either developed their own unique cobot platforms (n = 22), or authors who used off-the-shelf cobots, including Pepper (*n* = 3), Nao (*n* = 3), (see “Cobots used?” columns in Table 3). Six studies included came from similar authors/research teams, using the same or very similar cobotic platforms including two studies based in the United States (Dalal et al., 2018; Lundberg et al., 2022), two based in Thailand (Thamrongaphichartkul et al., 2020; Vongbunyong et al., 2021) and two based in the Netherlands (Boumans et al., 2018; Boumans et al., 2019 - see rows in gray in Table 3). We decided to include these works from similar authors due to the fact that discovering commitment to continuous study is itself a finding from our review, and a table summarizing the overlap is presented within the Supplementary Material.

**TABLE 3:** Product matrix of cobots effects on nurses’ work literature reviewed.

References		**Cobots used?			Target population					Design sample size	Research location	Robot application		Robot location			Robot features									Morphology			Technology readiness level (TRL)
		Off-the-shelf		Unique design	Patients			Healthcare workers									Mobile base	Functional arms	Natural language interface	Storage function	Clinical work								
		Pepper	NAO		Patients broadly	Elderly	Children	Healthcare workers broadly	Nurses			Social robots	Service & logistic robots	Near patients	Roaming healthcare facility	Other					Take patient vitals	Record survey data	Medication reminder and delivery	Record other patient data	Perform other nurse tasks	Mechanistic	Anthropomorphic	Zoomorphic	
Aiding nurses	Sun et al., 2023*			y				y		14	China		y			y		y	y						y	y			5
	Oishi et al., 2021*			y	y			y	y	Unknown	India		y		y		y						y		y	y			4
	Hossain et al., 2020*			y	y			y		20	India		y	y			y				y		y				y		4
	Yoo et al., 2021*			y				y		20	South Korea	y		y					y					y	y		y		5
	Boumans et al., 2018*	y			y			y		31	Netherlands	y	y	y			y	y	y			y					y		5
	Virgolin et al., 2021*			y				y		1	Sweden		y		y		y		y		y		y				y		5
	Bohlen et al., 2020*			y		y		y		unknown	Germany		y	y				y								y			3
	Liu et al., 2018*			y	y	y		y		5	Japan		y	y				y							y	y			5
	Mišeikis et al., 2020*			y	y			y		Unknown	Switzerland		y		y		y	y							y	y			9
	Rossi et al., 2022*		y				y			109	Italy	y		y			y		y								y		5
	Thamrongaphichartkul et al. (2020)			y	y			y		18	Thailand		y	y	y		y			y						y			8
	Guan et al. (2021)			y				y		Unknown	Malaysia		y		y		y			y					y	y			3
	Konara et al. (2020)			y				y		Unknown	Sri Lanka		y		y		y			y			y		y	y			2
	Farrier et al. (2019)		y				y			46	Canada	y		y			y	y	y						y		y		7
	Vongbunyong et al. (2021)			y	y			y		40	Thailand		y		y		y			y			y		y	y			8
	Antony et al. (2020)			y				y	y	Unknown	India		y		y		y				y		y	y		y			4
	Lundberg et al. (2022)			y					y	27	United States		y	y	y		y	y	y		y				y		y		6
	He et al. (2019)			y					y	Unknown	China		y			y		y							y	y			4
	van der Putte et al. (2019)	y			y				y	35	Netherlands		y	y			y	y	y					y			y		7
	Abubakar et al. (2020)			y	y				y	24	United States		y	y	y		y	y	y		y				y	y			5
	Dalal et al. (2018)			y					y	11	United States		y	y	y		y	y	y								y		4
	Miyake et al. (2019)			y	y				y	12	Japan	y	y	y					y					y	y		y		7
COVID	Sun et al., 2023*			y				y		14	China		y			y		y	y						y	y			5
	Oishi et al., 2021*			y	y			y	y	Unknown	India		y		y		y						y		y	y			4
	Hossain et al., 2020*			y	y			y		20	India		y	y			y				y		y				y		4
	Virgolin et al., 2021*			y				y		1	Sweden		y		y		y		y		y		y				y		5
	Prabhakar et al. (2020)			y	y			y	y	Unknown	India		y		y		y						y		y	y			4
	Toney et al. (2022)			y				y		1	India		y			y		y						y		y			4
	Dangi et al. (2021)			y				y		Unknown	India		y		y		y						y		y	y			3
Elder care and/or memory support	Bohlen et al., 2020*			y		y		y		unknown	Germany		y	y				y								y			3
	Mišeikis et al., 2020*			y	y			y		Unknown	Switzerland		y		y		y	y							y	y			9
	Boumans et al., 2019*	y			y			y		31	Netherlands	y	y	y			y	y	y			y					y		6
	Liu et al., 2018*			y	y	y		y		5	Japan		y	y				y							y	y			5
Social Support	Yoo et al., 2021*			y				y		20	South Korea	y		y					y					y	y		y		5
	Boumans et al., 2018*	y			y			y		31	Netherlands	y	y	y			y	y	y			y					y		5
	Rossi et al., 2021*		y				y			109	Italy	y		y			y		y								y		5
	Boumans et al., 2019*	y			y			y		31	Netherlands	y	y	y			y	y	y			y					y		6
	Ali et al. (2020)		y				y			86	Canada	y		y			y	y	y								y		7
	Do et al. (2021)			y	y	y		y		30	United States	y		y					y			y		y		y			7

The gray rows designate studies from the same authors/research teams, using the same or very similar robotic platforms.

*Indicates articles in more than one category.

**Because our final dataset only included robot platforms which are intended for direct human interaction and assistance to humans in some way, we consider all included applications as “cobots”, whether or not they are labeled as such by authors.

The intended users of the cobot included patients and healthcare workers. For patients, some studies did not specify beyond patients in general, (*n* = 13), which some were specifically targeted towards the elderly (*n* = 3) and children (*n* = 3). Many studies reported on cobots that were intended to help clinical workers in general (*n* = 19), while some explicitly named nurses as intended users (*n* = 9; see “Target population” columns in Table 3).

The types of cobot products described in the selected articles can be defined as cobot products that assist with nursing work (*n* = 22), during COVID (*n* = 7), for elder care and/or memory support (*n* = 4), and social support (*n* = 6) (see Table 3 for articles description).

Twenty-one articles were published by the design team, including 16 that were designed to aid nurses by automating or assisting in services (such as monitoring patient vitals, supporting patient transfer, etc.), and logistics (such as transporting medicine from nurses’ station to patient room, and delivery of things such as food; see “Cobot application” in Table 3). The country of origin for most cobot studies covered here is India (see “Design location” columns in Table 3). Interestingly, all the COVID-motivated articles (*n* = 7) were published by the design team majorly in India (*n* = 5) to provide services such as “Aido-Bot” used for cleaning and sterilization, as well as taking patient’s vitals (Hossain et al., 2020); aid logistics of items such as food, and medication for patients in isolation (Guan et al., 2021; Oishi et al., 2021). Of the 21 articles published by the design team, three were designed to aid elder care and/or memory support with the major goal of providing service and logistic support (see “Cobot application” columns), and three articles stated social support as the design team (design location are South Korea, Canada and the U.S.) motivation of design as an emotional support and companion for patients (see “Cobot application” columns).

**FIGURE 2:**
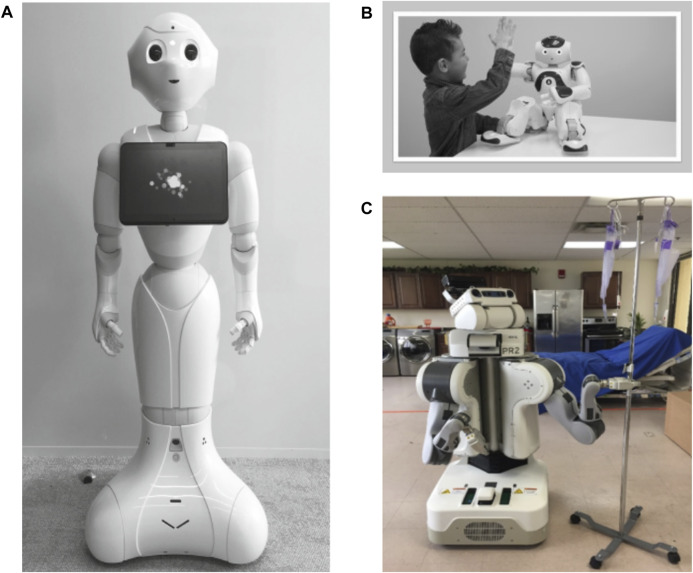
Examples of robots within included articles. **(A)** “Pepper”, the social humanoid robot (Boumans et al., 2019); **(B)** “MEDi”, an application of the humanoid robot NAO (Aldebaran, United Robotics Group, 2023) interacting with a child (Ali et al., 2020); **(C)** “PR2” robot during a fetching task (Dalal et al., 2018).

Various types of “Off-the-shelf” cobot products used in healthcare identified in the included articles are Pepper (*n* = 3), NAO (*n* = 1), and others including MEDi (*n* = 2) and “Personal Robot 2” (PR2) mobile cobot (*n* = 2; Dalal et al., 2018; Lundberg et al., 2022; see Figures 2A–C). Any other type of cobot that the article did not specify the type, or is a novel cobot designed by the research authors, or even a third-party design that is not any of the aforementioned three is classified as a unique design (*n =* 20). Examples of unique design cobots include “Lio”, a personal cobot assistant for routine tasks such as blood sample collection or mail delivery (Mišeikis et al., 2020), “Carver-Cap” cobot cart for logistics (Thamrongaphichartkul et al., 2020; Vongbunyong et al., 2021), adaptive cobotic nursing assistant (ARNA) platform for fetching objects for nurses and measuring patients’ temperatures (Lundberg et al., 2022), “ISOLDE”, a multimodal interactive mobile cobot for thermal measurement, and delivery of medicine and other object essential items (Virgolin et al., 2021), a social cobot used adapted for collecting patient-reported outcome measurement (Boumans et al., 2018; 2019) to mention a few (see “Cobots used” columns in Table 3; Supplementary Figures S1 for pictures of other cobot products).

Various target populations were described, including patients (e.g., Oishi et al., 2021; Hossain et al., 2020, etc.), clinical staff (e.g., Boumans et al., 2018; Guan et al., 2021, etc.), elderly (e.g., Liu et al., 2018; Bohlen et al., 2020), Healthcare workers (e.g., Virgolin et al., 2021; Konara et al., 2020; Dangi et al., 2021, etc.), caregivers (e.g., Bohlen et al., 2020; Miyake et al., 2020), children (includes., Farrier et al., 2019; Ali et al., 2020; Rossi et al., 2022), and nurses (includes, He et al., 2019; Abubakar et al., 2020; Anthony et al., 2020; Dalal et al., 2020; Miyake et al., 2020; Prabhakar et al., 2020; Oishi et al., 2021; Lundberg et al., 2022).

**FIGURE 3:**
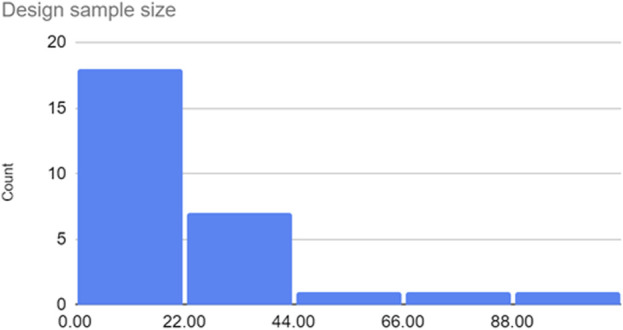
Design sample sizes within included articles (total participants).

**FIGURE 4:**
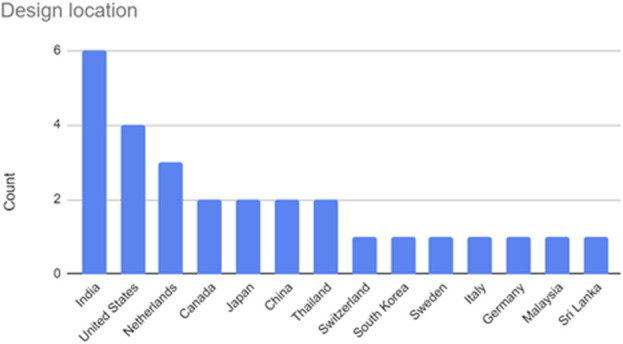
Design location counts within included articles.

The design sample size for all included articles was collected and Figure 3 illustrates the distribution of the sample sizes. Depending on the nature of the design study, nine articles did not provide design samples (e.g., Oishi et al., 2021; Prabhakar et al., 2020; Virgolin et al., 2021; see “Design sample size” columns in Table 3). The “Design location” was also described to indicate the country in which the research was undertaken or where the authors or affiliated institutions are located. This can be seen in Figure 4, and evidently, India (*n* = 6) tops the list, followed by the United States (*n* = 4), Netherlands (*n =* 3), and Canada, Japan, China, and Thailand (*n* = 2 each), then the other eight countries (*n* = 1 each).

### 3.2 Details on cobot platforms

This section provides the results of this research interest in the use of collaborative cobots to aid nurses in various healthcare settings, organized based on the following categories: cobot application, cobot location, cobot specifications, morphology, and Technology Readiness Level (TRL).

#### 3.2.1 Cobot applications in healthcare

The studies in the included literature described various applications of cobots in healthcare settings. Of the 28 included articles, the most common applications were for service and logistical tasks comprising twenty-three articles majorly for aiding nurse’s work (e.g., Anthony et al., 2020; Oishi et al., 2021; Sun et al., 2022), and assistance during the COVID-19 pandemic (e.g., Prabhakar et al., 2020; Dangi et al., 2021; Virgolin et al., 2022), then social cobots (n = 8) with three overlaps wherein the cobot performs both services and social functions (Boumans et al., 2018; Boumans et al., 2019; Miyake et al., 2020). The social cobots identified in the included articles were used to provide emotional support to patients especially children (e.g., Farrier et al., 2019; Ali et al., 2020; Rossi et al., 2022) and for providing information or taking clinical screening interviews (Do et al., 2021; Yoo et al., 2021; see “Cobot application” columns in Table 3). The literature review categorizes and examines these various applications, shedding light on the multifaceted roles cobots play in healthcare.

#### 3.2.2 Where is the cobot located?

The locations of the cobots were categorized into either “Near patients” or “Roaming healthcare facility” and “Other” for studies that did not report the location of the cobot. The most common locations were near patients as reported by about sixteen studies (Hossain et al., 2020; Yoo et al., 2021; Rossi et al., 2022). The majority of the cobots located near the patients were aiding nurses (*n* = 10) in performing tasks such as medication reminders (Hossain et al., 2020), or recording patients’ data (Miyake et al., 2020). All social support cobots (*n* = 6), and 75% of the elder care and/or memory support cobot (*n* = 3) were near the patients. Cobots that are roaming freely in the healthcare facilities (*n* = 13; Thamrongaphichartkul et al., 2020; Oishi et al., 2021) are majorly assisting nurses work (*n* = 9) such as a cobotic cart (Konara et al., 2020) for medication delivery, Carver-Cap cobot for logistics (Thamrongaphichartkul et al., 2020), mobile personal robot 2 (PR2), and adaptive cobotic nursing assistant (ARNA) platform for fetching objects for nurses and measuring patients’ temperatures (Dalal et al., 2018; Abubakar et al., 2020; Lundberg et al., 2022) to mention a few, while three studies did not report the location of the cobots (He et al., 2019; Toney et al., 2022; Sun et al., 2023; see “Cobot location” columns in Table 3).

#### 3.2.3 What types of cobot are included?

A couple of studies utilized existing commercial cobots like “Pepper” (*n* = 2), “NAO” (*n* = 1), or “MEDi” (*n* = 2), a modified version of NAO (Herald, 2015; Farrier et al., 2019). Beyond this, there were quite many studies that provided details about other cobot platforms such as “ISOLDE” (Virgolin et al., 2021) and “PR2” (Dala et al., 2018; Lundberg et al., 2022). The majority of the cobots were of “Unique design” (*n* = 20) developed by the design team such as “Lio”, a personal cobot assistant with a mobile base and a rechargeable battery that disinfects and detects elevated body temperature remotely (Mišeikis et al., 2020), “CARVER”, a cobotic cart with a mobile base and storage function used for medication delivery (Thamrongaphichartkul et al., 2020; Vongbunyong et al., 2021), or an “active monitoring bedside agent” used to prevent falls for older adults (Miyake et al., 2019; see “Cobot product used” columns in Table 3).

Regarding common features, most of these cobots were described as having a mobile base (*n* = 20). Half of the platforms had a functional robotic arm for manipulation (*n* = 14), and a natural language interface (*n* = 14). Some platforms had storage functionality (*n* = 4) or could record survey data (*n* = 3). A number of platforms could perform some nursing tasks such as medication reminder and delivery (*n* = 8), taking patient vitals (*n* = 5), or recording other patient data aside surveys or vitals data (*n* = 6). A number of studies reported the capability of performing “other” nursing tasks such as collecting oropharyngeal samples (Sun et al., 2023) or providing information to patients for a more self-directed hospital stay (Yoo et al., 2021). Other cobot features identified in the literature include “JAKA 141 ZU3” cobot with a DH-Robotics AG-95 gripper, single shot-multibox detector (SSD) that is used for sample collection (Sun et al., 2023), “Aido-Bot” used for vital monitoring with a specification combination of IR sensors, pulse oximeter sensor, and a mobile base (Hossain et al., 2020; see “Cobot features” columns in Table 3).

#### 3.2.4 Type of cobot morphology

In terms of morphology, many cobots had relatively mechanistic designs (*n* = 16) in which the designers did not try to create a humanlike or animal-like appearance (i.e., zoomorphic) and/or the design focused on utilitarian function instead of form but may choose to adopt a different appearance for future versions of the cobot. Some applications showed exposed electronics and wires which were likely due to the stage of the prototype and not the intention for the finalized product such as an autonomous mobile cobot (AMR) called “D-Bot” (Guan et al., 2021), or a cobotic cart called “CARVER” (Thamrongaphichartkul et al., 2020; Vongbunyong et al., 2021). The rest of the cobots in the included articles featured anthropomorphic designs (*n* = 12) such as the humanoid cobot “Pepper” from United Group Robotics (Boumans et al., 2018; Boumans et al., 2019), “NAO” and “MEDi” developed by Aldebaran from United Robotics Group (Farrier et al., 2019; Ali et al., 2020; Rossi et al., 2022), “PR2” (Dalal et al., 2018; Lundberg et al., 2022), or a multimodal interactive mobile cobot called “ISOLDE” (Virgolin et al., 2021; see “Morphology” columns in Table 3).

#### 3.2.5 Technological readiness

**FIGURE 5:**
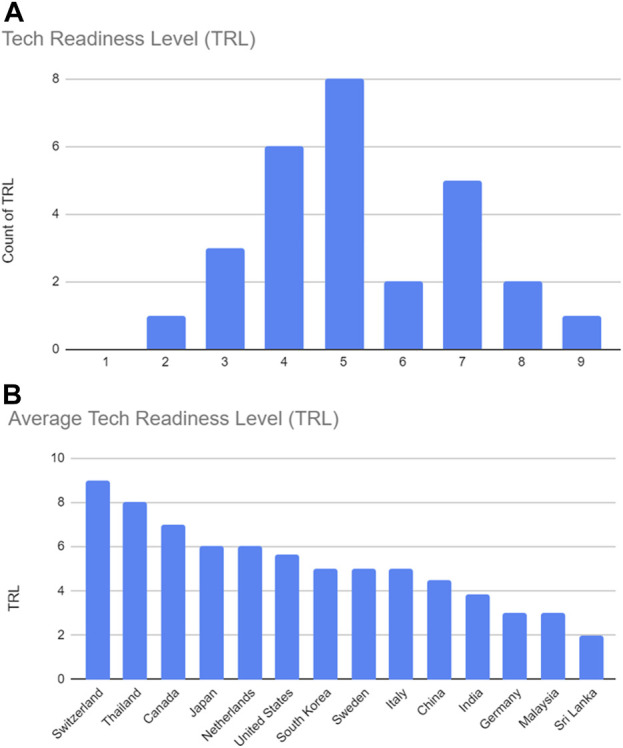
Technology Readiness Level (TRL). **(A)** TRL within included articles; **(B)** Average TRL per country within included articles.

The technology readiness level (TRL) reported for the cobots ranged from one to 9 (Manning, 2023), indicating prototypes to fully commercialized products. Most fell in the middle of the TRL range of 4 (*n* = 6) to 5 (*n* = 8), suggesting these cobots have moved from proof-of-concept to testing/validating components and/or breadboards in relevant environments, or simulation of environments that are realistic as possible. Five studies featured cobots at TRL 7, which means the system’ prototype is being demonstrated in a space environment, while only one study featured a cobot at TRL 9, suggesting the cobot has been implemented into the system and is being tagged “flight-qualified” and “flight proven” (Mišeikis et al., 2020), one study featured a cobot at TRL 2, indicating the cobot is at the early stage with little to no experimental proof-of-concept (Konara et al., 2020), and three studies featured cobots at TRL 3. The remaining studies (*n* = 4) featured cobots at either TRL 6 (*n* = 2) or TRL 8 (*n* = 2) that were in the early prototype to demonstration systems. This indicates most healthcare cobots are still at the testing/validation level (see Figures 5A, B and “Technology Readiness Level (TRL)” columns in Table 3).

In summary, these studies indicate a significant interest in the use of collaborative cobots to aid nurses in various healthcare settings, with a wide range of cobot types and applications. The Technology Readiness Level (TRL) varied across the studies, suggesting varying stages of development and implementation of these cobotic solutions.

Table 3 shows the resulting literature included after stages of the systematic review process using PRISMA guidelines (Page et al., 2021).

## 4 Discussion

With the increasing interest in utilizing cobots to support nursing work and enhance patient care, it is essential to examine the effectiveness and user perceptions of these machines in real-world healthcare settings. While previous studies have surveyed existing robotic platforms and explored future implications (e.g., Kangasniemi et al., 2019; Christoforou et al., 2020; Persson et al., 2022), this systematic review takes a deeper dive into the empirical evidence surrounding human interactions with cobots in laboratory and clinical environments. The main objective of this review is to establish a broad understanding of the impacts using cobots in healthcare settings has on nursing work and patient outcomes. This systematic review reveals several important findings regarding the current and future state of collaborative cobots designed to aid nursing work.

### 4.1 What does this review tell us about the current state of cobotics in healthcare?

#### 4.1.1 Current state

The literature shows that most healthcare cobots are patient-centered, with very few designed specifically to assist nurses. Additionally, most reported applications are based in India, with limited evidence from the U.S. In addition, a large proportion of COVID-related cobots emerged from India, representing a surge in development prompted by the pandemic. In terms of target populations, most existing cobots focus on patients in isolation or the elderly, delivering medications and other essentials, and providing emotional and companionship support, with minimal cobots designed for pediatric populations. Finally, the review highlights that the majority of market-ready technologies are based on off-the-shelf platforms like Pepper, NAO, MEDi, and PR2, rather than bespoke nurse-assistive designs.

#### 4.1.2 What future are we headed for?

This review reveals several trends that provide insight into the future landscape of collaborative cobots in healthcare. India accounted for the majority (21.4%) of published cobotics research wherein the majority (83.3%) were developed and deployed as COVID-specific cobots, illustrating significant investments in this area and the power of urgent demands driving innovation. However, evidence of nurse-assistive cobots in U.S. hospitals was generally strikingly lacking, despite the known adoption of general-purpose cobots like TUG Automated Robotic Delivery System by Aethon Inc. (Summerfield et al., 2011; Teng et al., 2022) and Moxi service cobot by Diligent Robotics (Bernardo et al., 2022; Tietze and Mcbride, 2020; Christensen et al., 2023). This indicates a gap between commercially-driven cobot purchases and academically-published scientific research, thereby highlighting a need for more research and reporting on real-world implementations rather than conceptual proposals as evidenced in Morgan et al. (2022).

The emphasis on care cobots is patient-centric rather than nurse-centric when the self-described purpose of most designs is to “aid nurses”, but instead they are replacing them. Cost analyses, maintenance planning, and hardware/software adaptability received little attention, though these factors are critical for long-term viability. Cybersecurity is also a major concern given the confidential patient data that healthcare cobots access (Guo, 2022; Whittaker, 2022). Without deliberation of these issues, seemingly promising cobots may fail prematurely.

With few exceptions, cobots appear to augment rather than automate nursing work. But truly nurse-centered designs remain rare. Overall, realizing the full potential of nurse-assistive cobots will require committed research addressing multi-year product lifecycles, versatile applications, and localized human-cobot collaboration. Rather than replace nurses, the ideal future sees collaborative robots efficiently handling rote tasks like materials transport, and documentation to free up nurses for high-value caregiving. This future will only emerge through deep partnerships between industry providers, healthcare organizations, and nurse stakeholders, acknowledging that successful adoption depends on far more than technological capabilities alone.

Overall, this review demonstrates significant gaps in the literature and market availability of collaborative robots tailored to aid nursing practice, particularly in the U.S. The findings suggest opportunities to apply human-centered design approaches engaging frontline nursing staff, to develop innovative cobots that can address nurses’ unique needs and workflows. Increased research commitments and industry partnerships focusing on nurse-assistive cobotics could help overcome existing barriers to adoption and implementation.

### 4.2 Limitations

This review has several limitations to note. While a systematic approach was used to survey relevant literature from the past 5 years, some relevant articles were likely missed. The structured search criteria also excluded articles on surgical, rehabilitation, and cobots not in a hospital setting that may involve nursing tasks. Additionally, details about target users and stakeholders were limited to what was explicitly stated in each article’s text. Some cobot applications probably involved additional target groups not reported on. Finally, as peer-reviewed publications were the sole focus, insights from industry white papers, perception or opinionated papers that do not include physical cobots, and commercial websites were not captured. This may skew findings away from widely adopted designs toward more conceptual proposals. Overall, the review provides a sampled cross-section of recent research, but cannot claim to be fully comprehensive nor capture all real-world developments in nurse-assistive cobotics. Expanded searches including non-peer-reviewed sources may reveal further insights.

### 4.3 Opportunities discovered through this review: future work

This review proffers a detailed overview and comparison of the current state of cobotics in healthcare and how they are being used to aid nursing tasks. This study complements other recent studies that have identified a greater need for innovation in the field of collaborative cobots in nursing (Frazier et al., 2019; Persson et al., 2022).

A major finding of this review is the lack of cobots designed specifically to collaborate with and aid nurses (32.14%, *n* = 9/28 articles) in hospital environments. The literature focuses predominantly on patient-centered (64.29%, *n* = 18/28 articles) care cobots which take an automation-oriented approach discordant with nurses’ needs. However, some nurse-assistive platforms like TUG (Summerfield et al., 2011; Teng et al., 2022) and Moxi (Tietze and Mcbride, 2020; American Hospital Association, 2022; Bonett, 2022). are already being used in U.S. hospitals, indicating a gap between commercial adoption and academic research.

These insights highlight significant opportunities to engage frontline nurses in the design of innovative cobots purpose-built to enhance nursing practice. Rather than replace nurses, collaborative robots could automate rote tasks like materials transport, and documentation to allow nurses to focus on high-value caregiving. Success will require committed research-practice partnerships leveraging nurses’ domain expertise to guide technical development (Kangasniemi et al., 2019; Tietze and Mcbride, 2020; Schulz-Schaeffer et al., 2023).

Additionally, this review revealed a lack of cobotics aimed at reducing administrative burdens, which are major contributors to clinician burnout. Logistical and documentation assistance represent promising applications aligned with nurses’ frustrations. Overall, realizing the full potential of nurse-centered design will require moving beyond automation toward meaningful human-cobot teaming that empowers nurses and improves satisfaction. The design process must bring nurse voices to the forefront to ensure resulting technologies integrate smoothly into existing clinical workflows.

## Data Availability

The original contributions presented in the study are included in the article/Supplementary Material, further inquiries can be directed to the corresponding authors.
