# Supplementary material for: A systematic review of collaborative robots for nurses: where are we now, and where is the evidence?
Source: Front Robot AI. 2024 Jun 5;11:1398140. doi: 10.3389/frobt.2024.1398140 (PMC11186321; doi:10.3389/frobt.2024.1398140)
Supplement: Supplementary file 1 [file DataSheet1.docx]

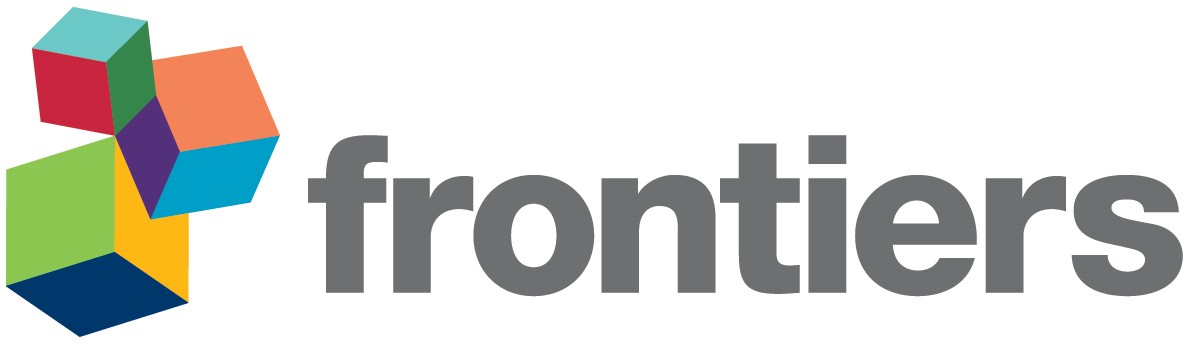


**SUPPLEMENTARY FIGURES**


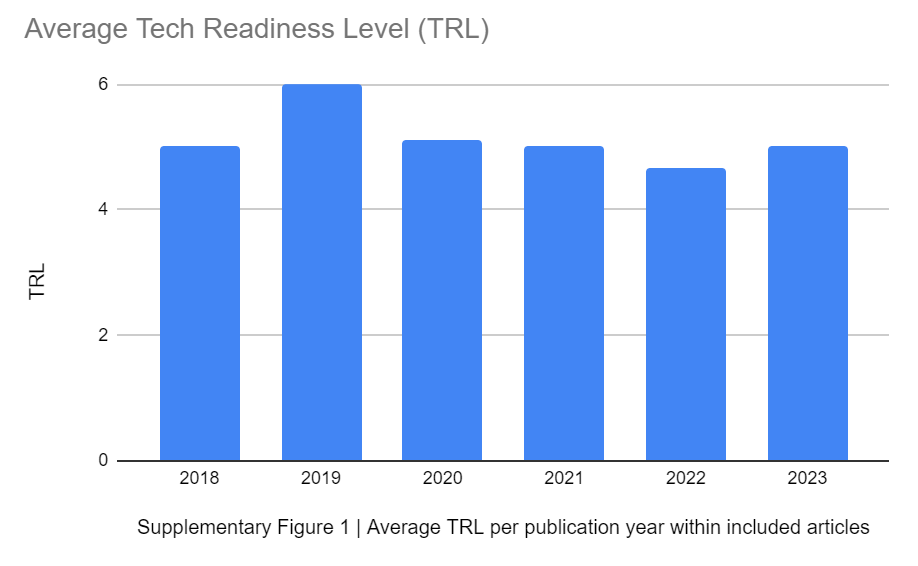


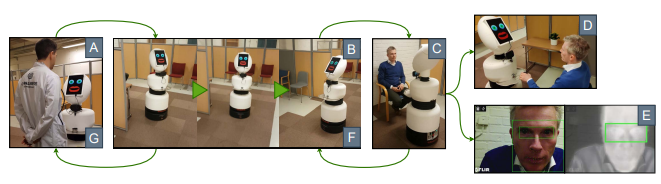


**Supplementary Figure 2** | “ISOLDE” robot in action (Virgolin et al., 2021)


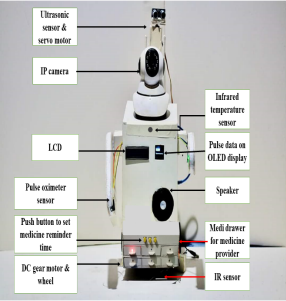


**Supplementary Figure 3** | “Aido-Bot” robot front view (Hossain et al., 2020)


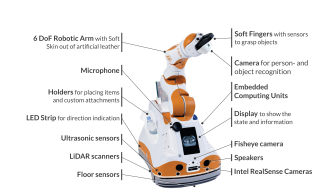


**Supplementary Figure 4** | “Lio” robot overview (Mišeikis et al., 2020)


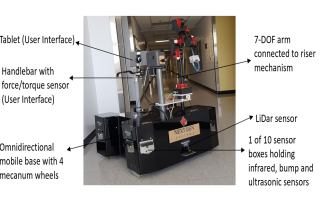


**Supplementary Figure 5** | “Adaptive Robot Nursing Assistant (ARNA)” robot overview (Abubakar et al., 2020)


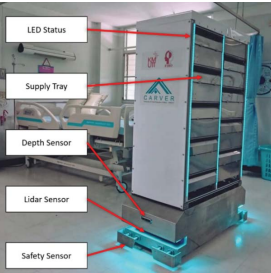


**Supplementary Figure** **6** | “CARVER” the autonomous mobile robot (AMR) overview (Thamrongaphichartkul et al., 2020)

**Supplementary Table 2** | Summary of studies from similar authors

| **Articles** | **Authors** | **Robotic platform** | **Functionality** | **Authors' assessment of similarity** |
| --- | --- | --- | --- | --- |
| Boumans et. al., 2018 | Roel Boumans,  Fokke van Meulen,  Koen Hindriks,  Mark Neerincx,  Marcel Olde Rikkert | Pepper v1.7 | For patient-reported outcome measures (PROM) data acquisition in older persons | 1) Exact same authors  2) Same robotic platform  3) Same functionality  4) Conference (n=1 participant) vs. Journal publication (n=31 participants) |
| Boumans et. al., 2019 | Roel Boumans,  Fokke van Meulen,  Koen Hindriks,  Mark Neerincx,  Marcel Olde Rikkert | Pepper v1.7 | For autonomous PROM data acquisition in older persons |  |
| Thamrongaphichartkul wt al., 2020 | Kitti Thamrongaphichartkul,  Nitisak Worrasittichai,  Teeraya Prayongrak,  Supachai Vongbunyong | Carver-AMR | For “delivering food” and “medical supplies” to individual patients | 1) Similar authors with one additional author who was not in the previous publication.  2) Similar robotic platform, adapted from previous study.  3) Robotic platform functionalities were adjusted and updated based on the hospital’s requirements from study 1. |
| Vongbunyong et al., 2021 | Supachai Vongbunyong,  Kitti Thamrongaphichartkul,  Nitisak Worrasittichai,  Aphisit Takutruea,  Teeraya Prayongrak | Carver-mini | For “item delivery” to individual patients |  |
| Dalal et. al., 2018 | Ankur Vipulkumar Dalal,  Ajinkya Mahadeo Ghadge,  Cody Lee Lundberg,  Jeongsik Shin,  Hakki Erhan Sevil,  Deborah Behan,  Dan O. Popa | Mobile robotic platform - PR2 | For object fetching tasks, specifically i) coke, ii) cereal, and iii) water bottle | 1) Similar authors but not the exact same authors.  2) Same robotic platform  3) More functionality to include more tasks in the second work. Hence, a progression of previous work.  4) Conference vs. journal paper, both describing similar but distinct research in great detail. |
| Lundberg et. al., 2022 | Cody Lee Lundberg,  Hakki Erhan Sevil,  Deborah Behan,  Dan O. Popa | Mobile robotic platform - PR2 | For:  i) Object fetching  ii) Temperature measurement  iii) Supporting patient while using the walker |  |

# REFERENCES

Abubakar, S., Das, S. K., Robinson, C., Saadatzi, M. N., Logsdon, M. C., Mitchell, H., Chlebowy, D., & Popa, D. O. (2020, August 1). *ARNA, a Service robot for Nursing Assistance: System Overview and User Acceptability*. IEEE Xplore.<https://doi.org/10.1109/CASE48305.2020.9216845>

Hossain, A., Hossain, E., Uddin, J., Sayeed, A., Uddin, A., Jinan, U. A., & Hossain, A. (2020, December 26). Design and Implementation of an IoT Based Medical Assistant Robot (Aido-Bot). *2020 IEEE International Women in Engineering (WIE) Conference on Electrical and Computer Engineering (WIECON-ECE)*.<https://doi.org/10.1109/wiecon-ece52138.2020.9397958>

Mišeikis, J., Caroni, P., Duchamp, P., Gasser, A., Marko, R., Mišeikienė, N., Zwilling, F., Castelbajac, C. de, Eicher, L., Früh, M., & Früh, H. (2020). Lio-A Personal Robot Assistant for Human-Robot Interaction and Care Applications. *IEEE Robotics and Automation Letters*, *5*(4), 5339–5346.<https://doi.org/10.1109/LRA.2020.3007462>

Thamrongaphichartkul, K., Worrasittichai, N., Prayongrak, T., & Vongbunyong, S. (2020, November 18). A Framework of IoT Platform for Autonomous Mobile Robot in Hospital Logistics Applications. *2020 15th International Joint Symposium on Artificial Intelligence and Natural Language Processing (ISAI-NLP)*.<https://doi.org/10.1109/isai-nlp51646.2020.9376823>

Virgolin, M., Bellone, M., Wolff, K., & Wahde, M. (2021). A Mobile Interactive Robot for Social Distancing in Hospitals. *2021 Fifth IEEE International Conference on Robotic Computing (IRC)*, 87–91.<https://doi.org/10.1109/irc52146.2021.00020>
